# Machine-learning scoring functions trained on complexes dissimilar to the test set already outperform classical counterparts on a blind benchmark

**DOI:** 10.1093/bib/bbab225

**Published:** 2021-06-24

**Authors:** Hongjian Li, Gang Lu, Kam-Heung Sze, Xianwei Su, Wai-Yee Chan, Kwong-Sak Leung

**Affiliations:** Chinese University of Hong Kong, Hong Kong; School of Biomedical Sciences, Chinese University of Hong Kong, Hong Kong; Bioinformatics Unit, Hong Kong Medical Technology Institute, Hong Kong; Chinese University of Hong Kong, Hong Kong; CUHK-SDU Joint Laboratory on Reproductive Genetics, School of Biomedical Sciences, Chinese University of Hong Kong, Hong Kong; Computer Science and Engineering in the Chinese University of Hong Kong, Hong Kong

**Keywords:** scoring function, machine learning, random forest, scoring power, binding affinity, blind benchmark

## Abstract

The superior performance of machine-learning scoring functions for docking has caused a series of debates on whether it is due to learning knowledge from training data that are similar in some sense to the test data. With a systematically revised methodology and a blind benchmark realistically mimicking the process of prospective prediction of binding affinity, we have evaluated three broadly used classical scoring functions and five machine-learning counterparts calibrated with both random forest and extreme gradient boosting using both solo and hybrid features, showing for the first time that machine-learning scoring functions trained exclusively on a proportion of as low as 8% complexes dissimilar to the test set already outperform classical scoring functions, a percentage that is far lower than what has been recently reported on all the three CASF benchmarks. The performance of machine-learning scoring functions is underestimated due to the absence of similar samples in some artificially created training sets that discard the full spectrum of complexes to be found in a prospective environment. Given the inevitability of any degree of similarity contained in a large dataset, the criteria for scoring function selection depend on which one can make the best use of all available materials. Software code and data are provided at https://github.com/cusdulab/MLSF for interested readers to rapidly rebuild the scoring functions and reproduce our results, even to make extended analyses on their own benchmarks.

## Introduction

**Figure 1:**
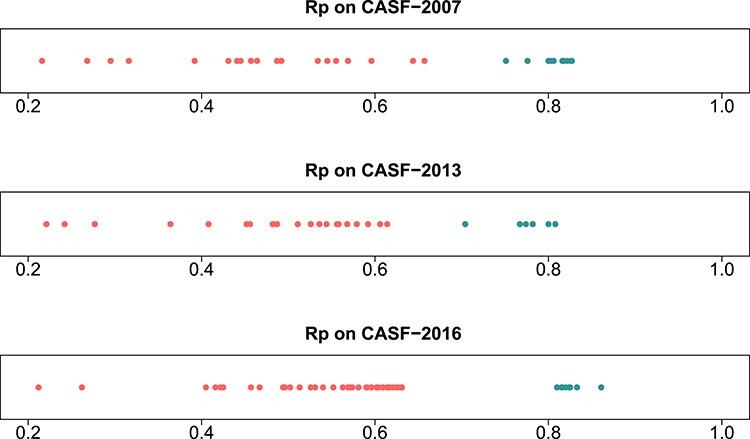
Performance of classical SFs (red dots) and ML-based SFs (green dots) on three CASF benchmarks. Each dot represents a SF. For instance, on CASF-2016 the best classical SF (i.e. X-Score) and the best ML-based SF (i.e. TopBP) obtained an Rp of 0.631 and 0.861, respectively. The raw values of this figure can be found in Supplementary Tables S1–S3.

In structural bioinformatics, the prediction of binding affinity of a small-molecule ligand to its intended protein is typically accomplished by a scoring function (SF). Before machine learning (ML)-based SFs were invented, classical SFs relied on linear regression of an expert-curated set of physiochemical descriptors to the experimentally measured binding affinities. ML-based SFs, however, bypass such prearranged functional forms and deduce a, often immensely, nonlinear model from the data. Two comprehensive reviews have discussed the outstanding performance of ML-based SFs over classical SFs in both scenarios of drug lead optimization [1] and virtual screening [2]. Here we focus on the former scenario, particularly the problem of binding affinity prediction. To illustrate to what extent the two types of SFs differ in predictive performance, we have compiled the results of 70 SFs and plotted Figure 1. It is now obvious that ML-based SFs are taking an apparent lead ahead of classical counterparts by a large margin.

One natural question to ask is whether the superiority of ML-based SFs originates from training on complexes similar to the test set. Exploring the influence of data similarity between the training and test sets on the scoring power of SFs has resulted in a chain of studies recently (Supplementary Table S4). In 2017, Li and Yang measured the training-test set similarity in terms of protein structures and sequences, and defined similarity cutoffs to construct nested training sets, with which they showed that random forest (RF)-based RF-Score lost to X-Score upon removal of training complexes whose proteins are highly similar to the CASF-2007 test proteins identified by structure alignment and sequence alignment [3]. However, in 2018 Li *et al.* found instead that RF-Score-v3 outperformed X-Score when 68% of the most similar proteins were deleted from the training set, suggesting that ML-based SFs owe a substantial portion of their performance to learning from complexes with dissimilar proteins to those in the test set. Unlike X-Score, RF-Score-v3 was able to keep learning with an increasing training set size, eventually becoming significantly more predictive (Rp = 0.800) than X-Score (Rp = 0.643) when the largest training set was used [4]. In addition to quantifying training-test set similarity by comparing protein structures or sequences, in 2019 these authors presented a new type of similarity metric based on ligand fingerprints. They observed that, regardless of which similarity metric was employed, training with a larger number of similar complexes did not boost the performance of classical SFs such as X-Score, Vina or Cyscore. On the other hand, XGB-Score, a SF utilizing extreme gradient boosting (XGBoost), was also shown to improve performance with more training data like RF-Score-v3 [5]. In 2020 Shen *et al.* further assessed 25 SFs, of which 21 are classical and four are ML-based. Six ML methods, namely RF, extra trees (ET), gradient boosting decision tree (GBDT), XGBoost, support vector regression (SVR) and k-nearest neighbor (kNN), were employed to build ML models using the features from the 25 SFs as well as their combinations. The results suggested that most ML-based SFs can learn not only from highly similar samples but also from dissimilar samples with varying magnitude [6]. The above studies all used CASF-2007 as the sole benchmark. Su *et al.* utilized CASF-2016 instead and calculated three similarity metrics considering protein sequence, ligand shape and binding pocket. Six ML algorithms were evaluated, including Bayesian ridge regression (BRR), decision tree (DT), kNN, multilayer perceptron (MLP), Linear SVR and RF. The RF model was found to possess the best learning capability and thus benefit most from the similarity between the training set and the test set, to which the three counterpart classical SFs, ChemScore, ASP and X-Score, were basically insensitive [7]. Sze *et al.* proposed a revised definition of structural similarity between a pair of training and test set proteins, introduced a different measure of binding pocket similarity, and benchmarked three classical SFs and four RF-based SFs on CASF-2013. They found that even if the training set was split into two halves and the half with proteins dissimilar to the test set was used for training, RF-based SFs still produced a smaller prediction error than the best classical SF, thus confirming that dissimilar training complexes may be valuable when allied with appropriate ML approaches and informative descriptors [8].

Here we have expanded the above six studies from the following perspectives. Firstly, we will demonstrate three examples to show that the method employed by four early works [3–6] for calculating structural similarity could be error prone, hence a revised method proposed lately [8] should be advocated. Secondly, in addition to CASF-2016, a blind evaluation was conducted too, where only data available until 2017 were used to construct the SFs that predict the binding affinities of complexes released by 2018 as if these had not been measured hitherto [1]. This blind benchmark offers a complementary interpretation of the results. Thirdly, while building ML-based counterparts of classical SFs, exactly the same descriptors were preserved. In this way, any performance difference must necessarily arise from the algorithmic substitution. The capability of feature hybridization was assessed too. Finally, we discussed the limitations of CASF and the inevitability of similarity contained in a large dataset, giving advice on the criteria of SF selection in a prospective setting.

## Materials and methods

**Figure 2:**
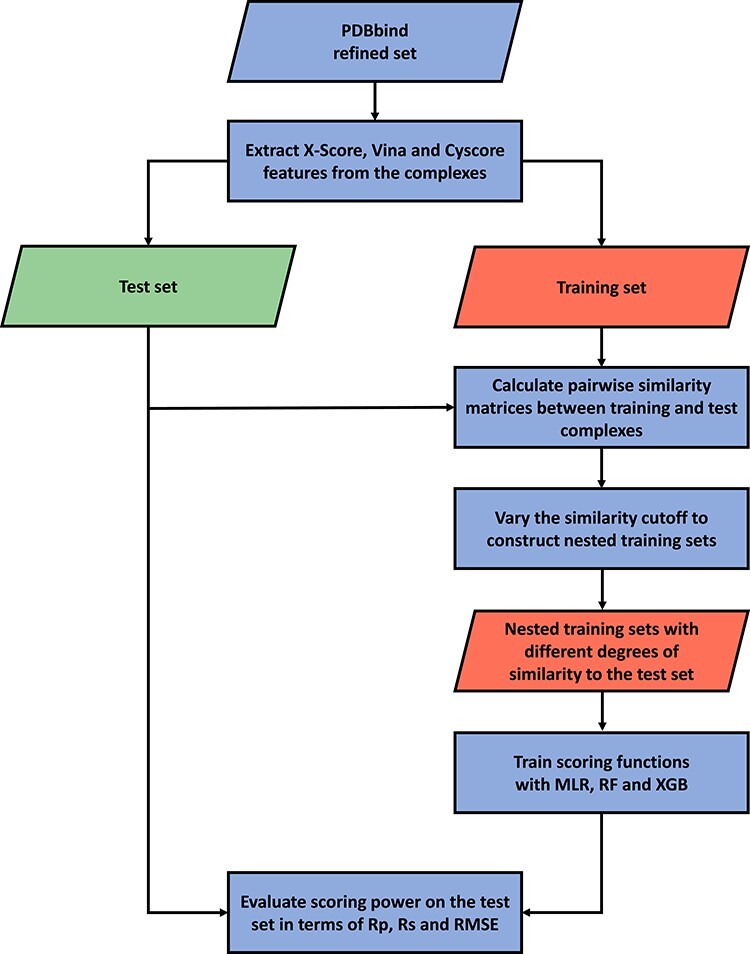
Workflow of training and test set similarity analysis.

Figure 2 presents the overall workflow of this study, which will be detailed in the following subsections.

### Performance benchmarks

The CASF benchmarks have been broadly employed to assess the scoring power of SFs. CASF-2016, the latest release, was utilized as the test set, which offers the crystal structures and binding affinities of 285 complexes sampled from 57 clusters. After removing the test complexes from the PDBbind v2016 refined set, the rest 3772 complexes were used as the training set. This was the same configuration as used in [7].

A recently proposed blind benchmark [1] mimicking the realistic process of structure-based lead optimization was adopted too, where the test set was constituted by 318 complexes from the PDBbind v2018 refined set not already included in the v2017 refined set. This test set is denoted Blind-2018 for short, on which 2 classical SFs and 3 ML-based SFs had been evaluated (Supplementary Table S5). It is totally different than CASF-2016 since they do not overlap, i.e. not a single complex coexists in both test sets. The 4154 complexes in v2017 served as the training set.

The scoring power of the considered SFs was measured by three commonly used quantitative indicators, namely Pearson correlation coefficient (Rp), Spearman correlation coefficient (Rs) and root mean square error (RMSE). A better performance is signified by higher values in Rp and Rs and lower values in RMSE.

### Similarity metrics

Obviously, the similarity of a training complex and a test complex can be measured in multiple ways, for example by their proteins, their ligands or their binding pockets. The approach by Sze *et al.* [8] was employed to calculate the similarity in terms of protein structure, ligand fingerprint and pocket topology.

In four early studies [3–6] the structural similarity between a pair of training and test set proteins was defined as the TM-score [9] calculated from the structure alignment program TM-align [10], which generates an optimized residue-to-residue alignment for comparing two protein chains whose sequences can be different. Nonetheless, TM-align is restricted to aligning single-chain monomer protein structures. Given that nearly half proteins of the PDBbind refined set contain multiple chains, each chain was extracted and compared, and the lowest pairwise TM-score was reported. This all-chains-against-all-chains approach could possibly step into the danger of misaligning a chain of a protein to an irrelevant chain in another protein. Three examples of misalignment are showcased in the Results section. To circumvent such risk, we switched to MM-align [11], which is specifically developed for aligning multiple-chain protein–protein complexes. It joins the multiple chains in a complex in every possible order and aligns them using a heuristic iteration of a modified Needleman–Wunsch dynamic programming algorithm with cross-chain alignments prohibited. Having been normalized by the test protein, the TM-score reported by MM-align was used to define the protein structure similarity. It falls in the range of (0,1]. A TM-score close to 0 indicates the two comparing proteins are substantially dissimilar, and a TM-score of 1 implies identity.

Although the protein structure similarity considers the entire protein structure in a global nature, the binding of a small-molecule ligand to its intended macromolecular protein is instead predominantly determined by the local environment of the binding pocket. Locally similar ligand-binding domains may be found in globally dissimilar proteins. For this sake, it is rational to supplement extra measures to reflect ligand similarity and pocket similarity. In terms of implementation, the similarity of the bound ligands of a pair of training and test complexes was defined as the Tanimoto coefficient of their ECFP4 fingerprints [12], whereas that of the binding pockets was described by the city block distance between their TopMap feature vectors encoding geometrical shape and atomic partial charges [13]. Note that the ligand fingerprint similarity also ranges from 0 to 1, but the Manhattan distance between TopMap vectors ranges from 0 to +∞. Therefore, the latter actually depicts the dissimilarity, rather than similarity, of the two comparing pockets. A value of 0 suggests identity, and a larger value implies larger difference. Taken together, the three similarity metrics provide distinct but complementary ways to quantify the degree of resemblance of the training set to the test set.

The pairwise similarity matrices can be found at the github repository. On CASF-2016 there are 285 complexes in the test set and 3772 complexes in the training set, hence 285×3772 pairwise similarity values in the matrix. On Blind-2018 there are 318×4154 similarity values.

### Training sets

**Figure 3:**
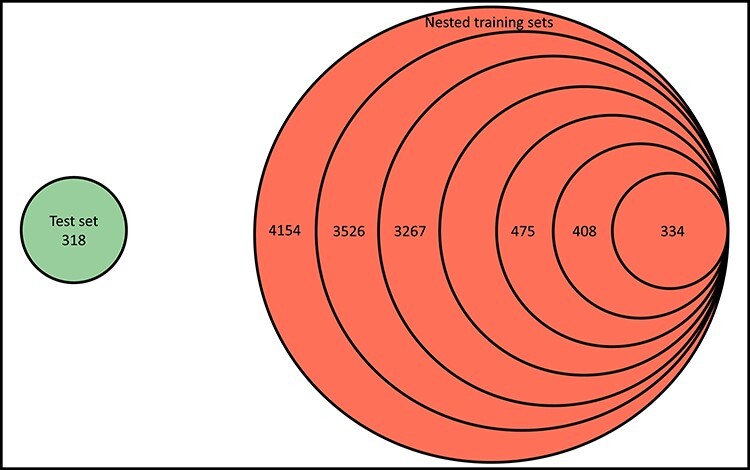
Venn diagram depicting the relationship between the test set and the nested training sets on Blind-2018. The numbers of training complexes 4154, 3526, 3267, …, 475, 408 and 334 correspond to the protein structure similarity cutoff values 1.00, 0.99, 0.98, …, 0.42, 0.41 and 0.40, respectively.

The original training set (OT) was split to a series of nested sets of training complexes with increasing degree of similarity to the test set in the following way. At a specific cutoff, a complex is excluded from the original full training set if its similarity to any of the test complexes is higher than the cutoff. In other words, a complex is included in the training set if its similarity to every test complex is always no greater than the cutoff [8]. Mathematically, for both protein structure and ligand fingerprint similarities whose values are normalized to [0, 1], a series of new training sets (NTs) were created by gradually removing complexes from the OT according to varying cut-off values given a fixed test set (TS):

(1) \begin{equation*} {NT}_{ds}^s(c)=\left\{\ {p}_i\ |\kern0.50em {p}_i\in OT\ and\ \forall{q}_j\in TS,s\left({p}_i,{q}_j\right)\le c\ \right\} \end{equation*}

where *c* is the cutoff; ${p}_i$ and ${q}_j$ represent the *i*th and *j*th complexes from OT and TS, respectively; and $s({p}_i,{q}_j)$ is the similarity between ${p}_i$ and ${q}_j$. By definition, ${NT}_{ds}^s(1)= OT$. When the cutoff varies from 0 to 1, nested sets of training complexes with increasing degree of similarity to the test set were constructed. For instance, with Blind-2018 as the test set, the protein structure similarity cutoff starts at 0.40 and ends at 1.00 with a step size of 0.01, thereby generating 61 nested training sets. Figure 3 shows a Venn diagram characterizing the relationship between the test set and the nested training sets. The test set does not overlap any of the constructed training sets, but the latter overlap each other, e.g. setting the cutoff to 0.40, 0.41 and 0.42 results in three training sets ${NT}_{ds}^s(0.40)$, ${NT}_{ds}^s(0.41)$ and ${NT}_{ds}^s(0.42)$ with 334, 408 and 475 complexes, respectively, and the latter is a superset of the former, i.e. ${NT}_{ds}^s(0.40)\subseteq{NT}_{ds}^s(0.41)\subseteq{NT}_{ds}^s(0.42)$. When the cutoff reaches 1.00, all the 4154 training complexes will be included in ${NT}_{ds}^s(1.00)$. Likewise, the ligand fingerprint similarity starts at 0.50 and ends at 1.00 with a step size of 0.01, thereby creating 51 nested training sets.

In the case of pocket topology, since the values indicate dissimilarity instead of similarity and they fall in the range of [0, +∞], a slightly different definition is required:

(2) \begin{equation*} {NT}_{ds}^d(c)=\left\{\ {p}_i\ |\kern0.50em {p}_i\in OT\ and\ \forall{q}_j\in TS,d\left({p}_i,{q}_j\right)\ge c\ \right\} \end{equation*}

where d$({p}_i,{q}_j)$ is the dissimilarity between ${p}_i$ and ${q}_j$. Likewise, ${NT}_{ds}^d(0)= OT$ and ${NT}_{ds}^d(+\infty )=\varnothing$. When the cutoff steadily decreases from +∞ to 0, nested training sets with increasing degree of similarity to the test set were generated. The pocket topology dissimilarity cutoff starts at 10.0 and ends at 0.0 with a step size of 0.2, thus generating 51 nested training sets.

Analogously, the opposite direction was also considered, where nested sets of training complexes with increasing degree of dissimilarity to the test set were built as follows:

(3) \begin{equation*} {NT}_{sd}^s(c)=\left\{\ {p}_i\ |\kern0.50em {p}_i\in OT\ and\ \exists{q}_j\in TS,s\left({p}_i,{q}_j\right)>c\ \right\} \end{equation*}

(4) \begin{equation*} {NT}_{sd}^d(c)=\left\{\ {p}_i\ |\kern0.50em {p}_i\in OT\ and\ \exists{q}_j\in TS,d\left({p}_i,{q}_j\right)<c\ \right\} \end{equation*}

The former applies to protein structure and ligand fingerprint similarities, and the latter applies to pocket topology dissimilarity. By definition, ${\forall}{c},{{NT}}_{{d}{s}}^{{d}}({c})\cup{{NT}}_{{sd}}^{{d}}({c})={OT},{{NT}}_{{d}{s}}^{{d}}({c}){\cap}{{NT}}_{{sd}}^{{d}}({c})={\varnothing}$. The corresponding number of training complexes given a cutoff can be found at the github repository.

### Scoring functions

Classical SFs undertaking multiple linear regression (MLR) were compared to their ML-based variants. X-Score [14] v1.3 was chosen to be a representative of classical SFs because on CASF-2016 it resulted in the highest Rp performance among a panel of 33 linear SFs (Figure 1), many of which are implemented in commercial software [15]. It also performed the best on CASF-2013 and the second best on CASF-2007 among classical SFs. It is a consensus of three parallel scores considering four intermolecular descriptors: van der Waals interaction, hydrogen bonding, hydrophobic effect and deformation penalty. These constituent SFs simply differ in the calculation of hydrophobic effect. To create MLR::Xscore, the three parallel SFs were independently trained with coefficients calibrated on the nested training sets, and then averaged to produce a consensus score. To make an ML-based counterpart, the same six features were reused but MLR was replaced by RF, thus creating RF::Xscore.

Given that X-Score dated back in 2002, two recent SFs, AutoDock Vina [16] v1.1.2 and Cyscore [17] v2.0.3, were also selected to represent classical SFs. Vina was selected because it is highly cited and widely used. Cyscore was selected because it yielded the highest Rp among 19 linear SFs on CASF-2007 (Figure 1). Cyscore is a strict MLR model composed of four intermolecular features: hydrophobic free energy, van der Waals interaction energy, hydrogen-bond energy and the ligand’s entropy. Vina is a quasi-MLR model where the weighted sum of five empirical terms is normalized by a conformation-independent ligand-only feature codenamed Nrot, which implies the degree of conformational freedom of the ligand. To imitate this specialty, the original weight for Nrot was adopted without recalibration while building MLR::Vina. A side effect is that its RMSE performance will become unreliable, from which we will avoid drawing conclusions. The RF counterparts RF::Vina [18] and RF::Cyscore [19] were generated with the same set of six descriptors from Vina and four descriptors from Cyscore, respectively. Supplementary Table S6 summarizes the molecular features. The full feature set for all the complexes can be found at the github repository.

Additionally, the features from X-Score, Vina and Cyscore were combined and fed to RF and XGBoost, thereby producing RF::XVC and XGB::XVC (taking the first letter of each SF). The purpose was twofold: to explore by how much the mixed descriptors would contribute to the predictive accuracy, and to compare between RF and XGB which are both tree-based ML algorithms.

## Results and discussion

### Misalignment caused by the all-chains-against-all-chains approach

**Figure 4:**
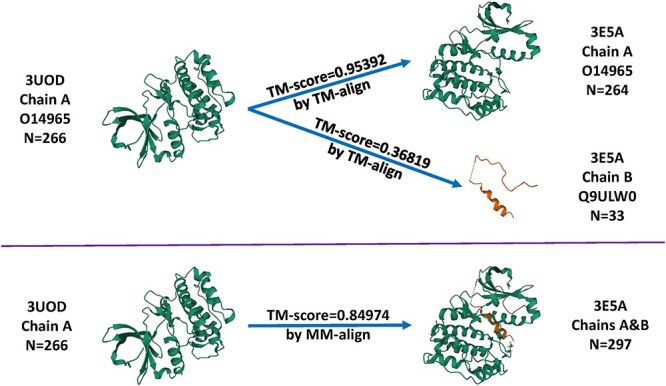
Comparison between TM-align and MM-align when structurally aligning the 3UOD entry in the training set and the 3E5A entry in the test set of CASF-2016.

We first show that the all-chains-against-all-chains method employed in four early works [3–6] could lead to misalignment. For example, the 3E5A entry in the CASF-2016 test set describes a crystal structure of aurora kinase A (chain: A; sequence length: 264) in complex with a small-molecule inhibitor and the targeting protein for Xklp2 (chain: B; sequence length: 33); the 3UOD in the training set describes another crystal structure of aurora kinase A (chain: A; sequence length: 266) in complex with another small-molecule inhibitor. A reasonable alignment should be aligning 3UOD chain A to 3E5A chain A because they both describe the main target protein. Were the all-chains-against-all-chains approach employed, every chain would be extracted and aligned, and the lowest pairwise TM-score would be used. In this case (Figure 4), aligning 3UOD chain A to 3E5A chain A would produce a TM-score of 0.95392 (Supplementary Material S1) whereas aligning 3UOD chain A to 3E5A chain B would produce a TM-score of 0.36819 (Supplementary Material S2), and therefore the latter would be reported as the similarity score between 3UOD and 3E5A, which apparently does not make sense. In contrast, with MM-align, aligning 3UOD to 3E5A resulted in a TM-score of 0.84974, which seems rational. Looking at the output of MM-align (Supplementary Material S3), one may find that 3UOD chain A was in fact aligned to 3E5A chain A as expected, without enforcing an alignment on 3UOD chain B to 3E5A chain A. Hence, the all-chains-against-all-chains approach underestimates the similarity by 0.48155 in this example. Although one can still use TM-align to align proteins after manually joining the chains, this would lead to suboptimal outcome with unphysical cross-chain alignments, which are prohibited in MM-align.

**Figure 5:**
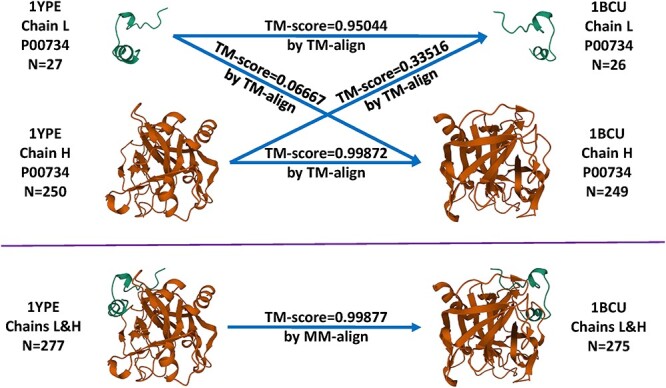
Comparison between TM-align and MM-align when structurally aligning the 1YPE entry in the training set and the 1BCU entry in the test set of CASF-2016.

Another example is about aligning the 1YPE entry in the training set to the 1BCU entry in the test set of CASF-2016 (Figure 5). Both entries comprise two chains of thrombin, but of different lengths. The L chain contains 27 and 26 residues for 1YPE and 1BCU, respectively, whereas the H chain contains 250 and 249 residues. Were the all-chains-against-all-chains approach employed, four TM-score values would be generated by TM-align and the lowest of them, 0.06667, would be reported as the similarity score between 1YPE and 1BCU. This score, however, measures the degree of structural agreement between a thrombin light chain of 27 residues and a thrombin heavy chain of 249 residues. As a result, it turns out to be understandably low. In contrast, MM-align reported a TM-score of 0.99877, which seems reasonable because all the four chains describe the crystal structure of thrombin with the same UniProt ID. Hence, the approach by TM-align underestimates the similarity by 0.9321 in this case. An analogous example is aligning the training set entry 1GJ8 to the test set entry 1C5Z, where MM-align reported 0.99419 but the all-chains-against-all-chains approach reported 0.03351, equivalent to an underestimation of as much as 0.96068. Remind that structures with a TM-score higher than 0.5 assume generally the same fold [10]. Hence, such underestimation could regard structures supposed to be of the same fold wrongly to be of distinct folds.

Overall, such bias is not a frequent phenomenon. Among all the 1 075 020 (=285×3772) pairwise similarities of CASF-2016, the portion where the difference in TM-score computed by the two approaches lies within 0.1 is 83%. This percentage is 85% on Blind-2018 over its 318×4154 similarities. When the difference threshold is relaxed to 0.2, the percentage increases to 95% for CASF-2016 and 98% for Blind-2018, indicating a high degree of agreement by the two approaches in most cases, and thus the conclusions in the four early studies employing TM-align are unlikely to deviate much. Despite the consistency, we advocate the more robust approach by MM-align, first introduced by Sze *et al.* [8].

### Skewed distribution of training complexes over (dis)similarity cutoffs

**Figure 6:**
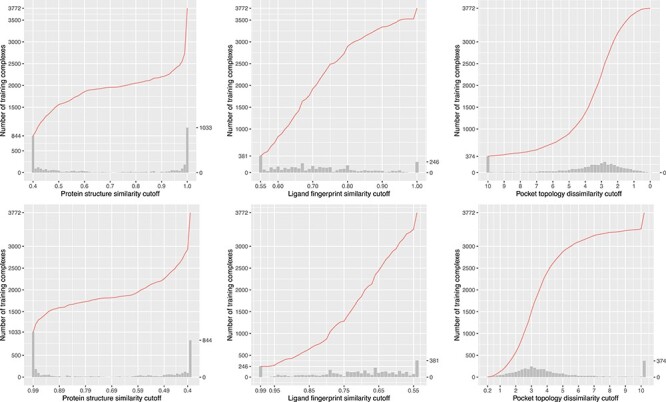
Number of training complexes (the red curve) against protein structure similarity cutoff (left column), ligand fingerprint similarity cutoff (center column) and pocket topology dissimilarity cutoff (right column) to the CASF-2016 test set in two directions, either starting from a small training set comprising complexes most dissimilar to the test set (top row; the ds direction defined by ${NT}_{ds}^s$ or ${NT}_{ds}^d$) or starting from a small training set comprising complexes most similar to the test set (bottom row; the sd direction defined by ${NT}_{sd}^s$ or ${NT}_{sd}^d$). At the top row, the histograms plot the number of additional complexes that will be added to a larger set when the protein structure similarity cutoff is incremented by a step size of 0.01 (left), when the ligand fingerprint similarity cutoff is incremented by 0.01 (center), or when the pocket topology dissimilarity cutoff is decremented by 0.2 (right). At the bottom row, the histograms plot the number of additional complexes that will be added to a larger set when the protein structure similarity cutoff is decremented by a step size of 0.01 (left), when the ligand fingerprint similarity cutoff is decremented by 0.01 (center), or when the pocket topology dissimilarity cutoff is incremented by 0.2 (right). Hence the number of training complexes referenced by an arbitrary point of the red curve is equal to the cumulative summation over the heights of all the bars of and before the corresponding cutoff. By definition, the histograms of the three subfigures at the bottom row are identical to the histograms at the top row after being mirrored along the median cutoff, but the cumulative curves are certainly different. The raw values of this figure are available at https://github.com/cusdulab/MLSF.

**Figure 7:**
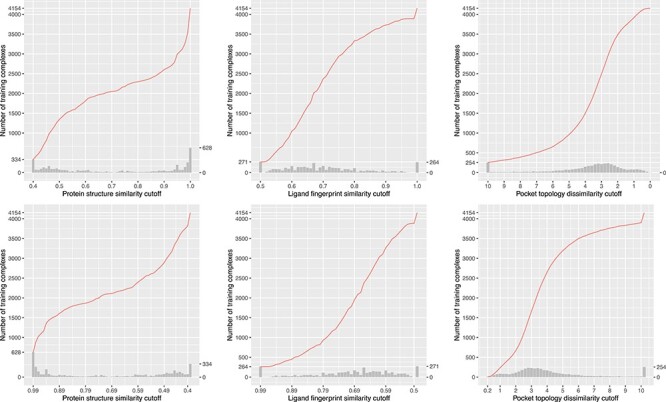
Same as Figure 2 but substituting the Blind-2018 test set.

We plotted the number of training complexes against the cut-off values of the three similarity metrics where the test set was CASF-2016 (Figure 6) or Blind-2018 (Figure 7), in order to show that training complexes are far from being evenly distributed. In reality, the distribution of training complexes under the protein structure similarity metric is skewed, e.g. 628 training complexes have a test set similarity greater than 0.99 (Figure 7, top left subfigure). The rightmost bar alone already accounts for 15% of the OT of 4154 complexes. Incrementing the cutoff by only 0.01 from 0.99 to 1.00 will include 15% additional training complexes. On the contrary, just 1.6% additional training complexes will be included when the cutoff is incremented by the same step size from 0.95 to 0.96. Hence, it is not surprising to observe a substantial performance boost from raising the cutoff by merely 0.01 if it is already at 0.99. This is more apparent in the CASF-2016 benchmark (Figure 6, left column), where for as many as 1033 training complexes (accounting for 27% of the full 3772 complexes) their test set similarities fall in the range of (0.99, 1]. Thus one would reasonably anticipate a sharp leap in Rp performance of ML-based SFs in this particular range, as seen in previous studies [3, 8] and this study (see the next subsection).

Such skewness can also be spotted, though less apparent, in the distribution under ligand fingerprint similarity (Figures 6 and 7, center column), where 6.5% and 6.4% training complexes have a test set similarity greater than 0.99 under the CASF-2016 and Blind-2018 benchmarks, respectively. The distribution against pocket topology dissimilarity (Figure 7, right column), nevertheless, seems relatively uniform, with just 0.1% complexes falling in the range of [0, 0.2) and just 6% in the range of [10, +∞). This pocket topology dissimilarity metric therefore constitutes a useful tool to explore the impact of data similarity on the scoring power of SFs with training set size not so skewed toward both ends of cutoff.

**Figure 8:**
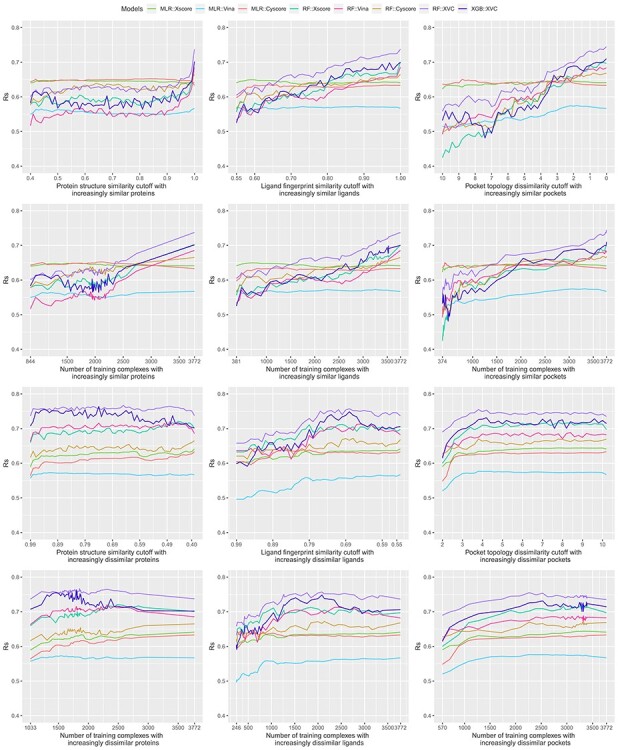
Rp performance of three classical SFs (MLR::Xscore, MLR::Vina and MLR::Cyscore) and five ML-based SFs (RF::Xscore, RF::Vina, RF::Cyscore, RF::XVC and XGB::XVC) on the CASF-2016 benchmark when they were calibrated on nested training sets generated with protein structure similarity (left column), ligand fingerprint similarity (center column) and pocket topology dissimilarity (right column). The first row plots the performance against cutoff, whereas the second row plots essentially the same result but against the associated number of training complexes instead. Both rows present the result where the nested training sets were initially formed by complexes most dissimilar to those in the test set and then gradually expanded to incorporate similar complexes as well (i.e. the ds direction). The bottom two rows depict the performance in a reverse similarity direction where training complexes similar to those in the test set were exploited initially and then dissimilar complexes were progressively included as well (i.e. the sd direction). The raw values of this figure are available at https://github.com/cusdulab/MLSF.

**Figure 9:**
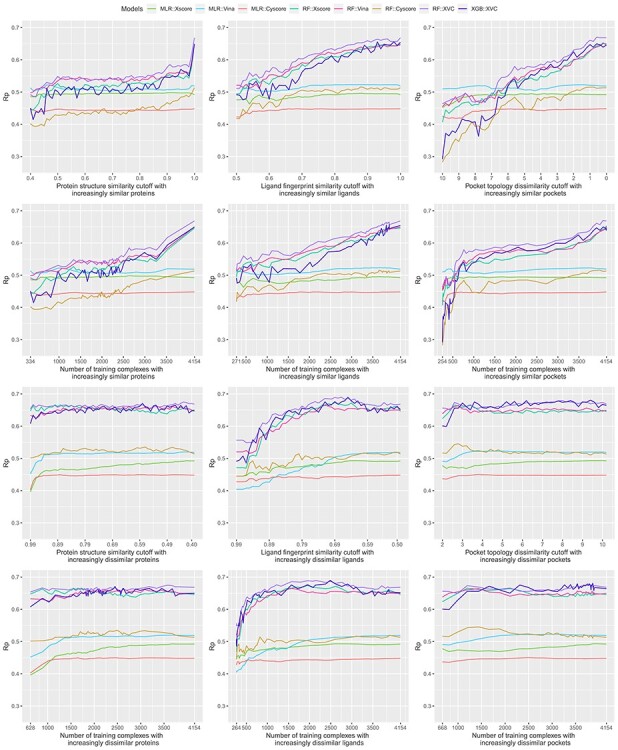
Same as Figure 4 but substituting the Blind-2018 benchmark.

**Figure 10:**
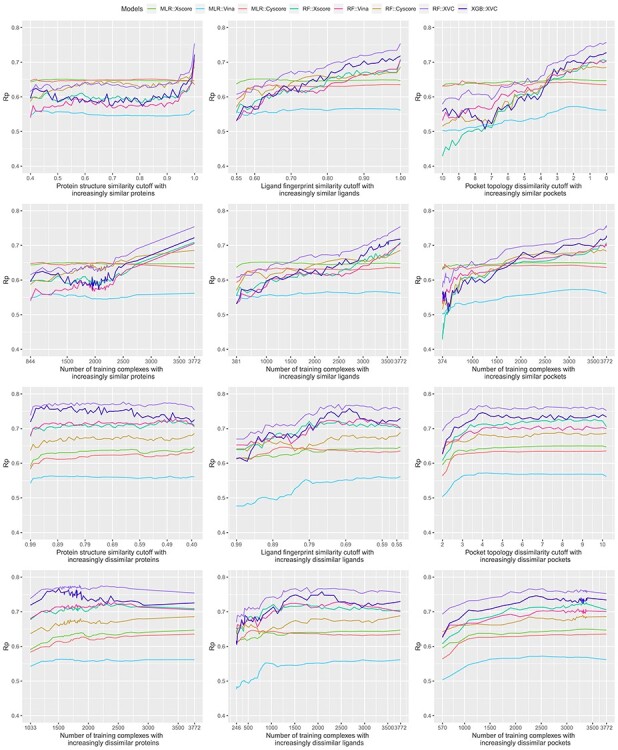
Same as Figure 4 but substituting Rs performance.

**Figure 11:**
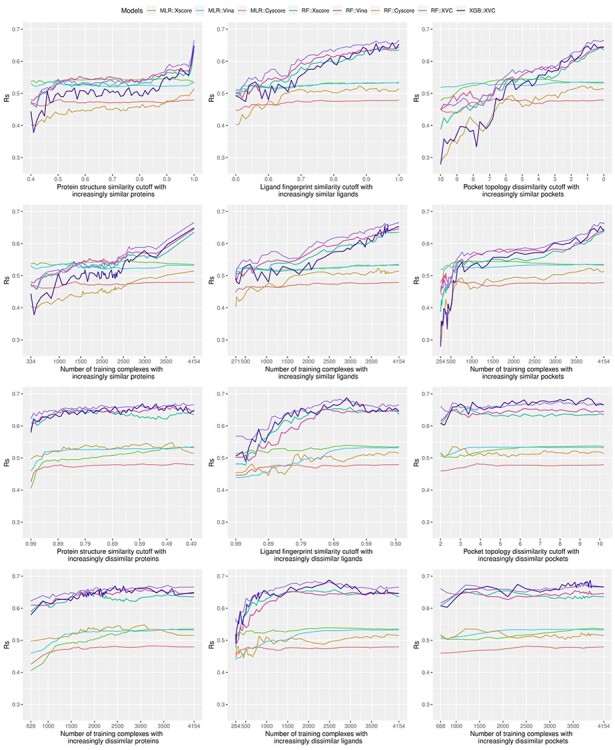
Same as Figure 6 but substituting the Blind-2018 benchmark.

**Figure 12:**
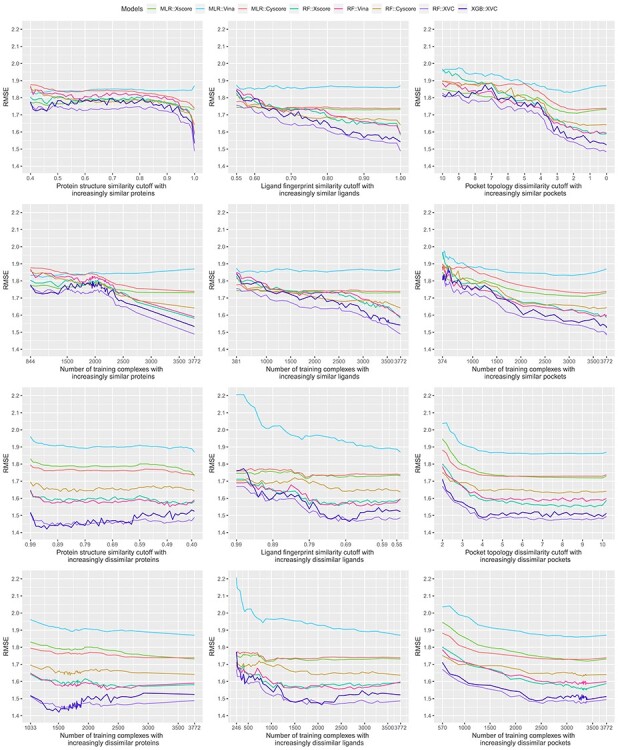
Same as Figure 4 but substituting RMSE performance.

**Figure 13:**
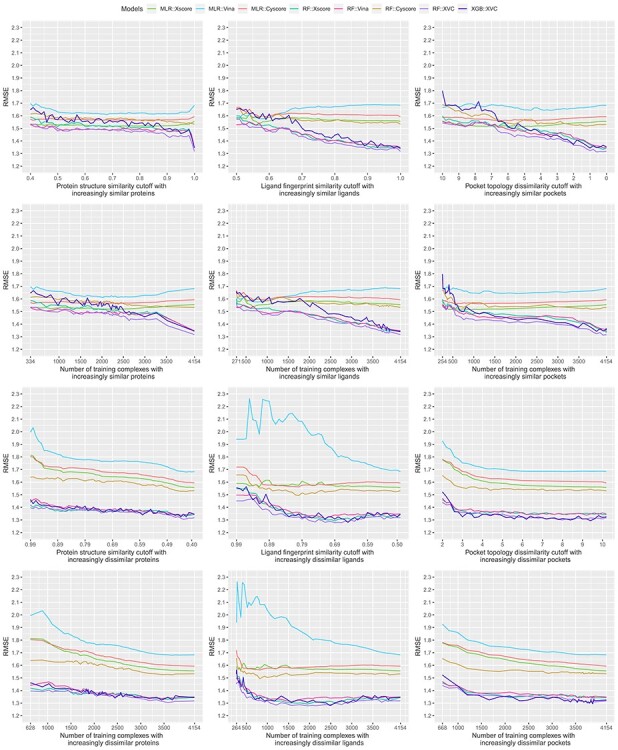
Same as Figure 8 but substituting the Blind-2018 benchmark.

Bearing in mind the non-even distributions described above, we retrained the three classical SFs (MLR::Xscore, MLR::Vina and MLR::Cyscore) and the five ML-based SFs (RF::Xscore, RF::Vina, RF::Cyscore, RF::XVC and XGB::XVC) on the nested training sets generated with protein structure similarity, ligand fingerprint similarity and pocket topology dissimilarity, and plotted their scoring performance on CASF-2016 (Figures 8, 10 and 12) and Blind-2018 (Figures 9, 11 and 13) in a consistent scale against either cutoff or number of training complexes in two similarity directions, i.e. the ds direction specified by ${NT}_{ds}^s$ in Equation 1 or by ${NT}_{ds}^d$ in Equation 2, and the sd direction specified by ${NT}_{sd}^s$ in Equation 3 or by ${NT}_{sd}^d$ in Equation 4.

### Sharp leap in scoring power of ML-based SFs benefiting from sufficient number of similar complexes for training

**Figure 14:**
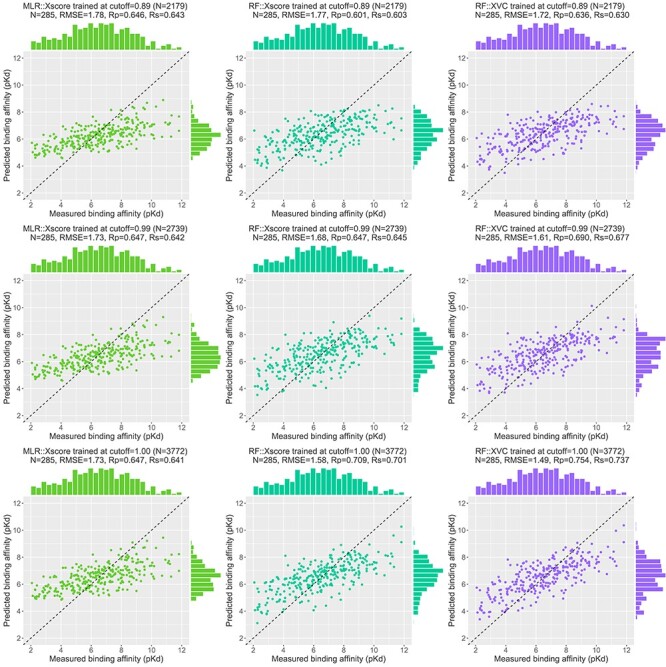
Scatter plots of predicted and measured binding affinities on CASF-2016 (*N* = 285 test complexes). Three SFs are compared: MLR::Xscore (left column), RF::Xscore (center column) and RF::XVC (right column), trained at three different cutoffs: 0.89 (1st row), 0.99 (2nd row) and 1.00 (3rd row), corresponding to 2179, 2739 and 3772 training complexes, respectively. The cutoff 0.89 represents the crossing point where RF::Xscore started to produce lower RMSE values than MLR::Xscore, indicating that although ML-based SFs did not outperform classical SFs in terms of Rp after removal of training complexes highly similar to the test set, it is not true when RMSE is considered. The cutoff 0.99 represents a training set without complexes highly similar to the test set (recall the skewed distribution in Figure 6), for comparison to the cutoff 1.00 in order to demonstrate the sharp leap effect in Rp and Rs and the sharp drop effect in RMSE.

Looking at the top left subfigure of Figure 9, which plots Rp performance on Blind-2018 versus protein structure similarity cutoff, not unexpectedly sharp leaps are observed within the rightmost range of (0.99, 1] for all the five ML-based SFs. For instance, the Rp notably increased by 0.067 (i.e. from 0.579 to 0.646) for RF::Xscore, by 0.065 for RF::Vina, by 0.051 for RF::XVC, as well as by 0.056 for XGB::XVC. This is also true on CASF-2016 (Figure 8, top left subfigure; Figure 14, bottom two rows). Likewise, sharp leaps in Rs (Figures 10 and 11) and sharp drops in RMSE (Figures 12 and 13) are observed for ML-based SFs too (e.g. the RMSE of RF::Xscore decreased by 0.11 from 1.45 to 1.34 on Blind-2018, versus a reduction of 0.05 in RMSE of the same SF within the second rightmost range of (0.98, 0.99] and a reduction of just 0.01 within the fourth rightmost range of (0.96, 0.97]) because, as explained in the subsection above, this particular range comprises as many as 27% and 15% training complexes of the CASF-2016 and Blind-2018 benchmarks, respectively, suggesting that ML-based SFs are effective at learning from training complexes highly similar to the test set.

Under the ligand fingerprint similarity metric (Figures 8–13, top center subfigure), such Rp and Rs performance leaps and RMSE drops for ML-based SFs within the rightmost range can be spotted too (e.g. the Rp of RF::XVC increased by 0.011 from 0.657 to 0.668 and its RMSE decreased by 0.02 from 1.34 to 1.32 on Blind-2018), though not as sharp because the distribution of training complexes is not as skewed (Figures 6 and 7, center column). Under the pocket topology dissimilarity metric (Figures 8–13, top right subfigure) where the distribution is relatively uniform (Figures 6 and 7, right column), no leaps in Rp and Rs or drops in RMSE are visually detectable (e.g. the difference in Rp of RF::XVC is less than 0.001 and the difference in RMSE is less than 0.01 on Blind-2018, thus not perceivably observable). These findings confirm that the remarkable performance gain obtained by ML-based SFs within this range of cutoff is not exclusively due to the high similarity, but also attributed to the considerable increase of training set size.

### Learning capability of ML-based SFs as an advantage over classical SFs

All the five ML-based SFs exhibited learning capability to some extent, proliferating performance with larger sets of increasingly similar training samples. RF::XVC, empowered by its combination of features from three SFs, performed better than their individual RF-based SFs on Blind-2018 (Figure 9), CASF-2016 (Figure 8) and CASF-2013 [8]. The runner up was RF::Vina, followed by RF::Xscore and lastly by RF::Cyscore, which somehow underperformed on Blind-2018, CASF-2013 [8] and CASF-2007 [6]. Despite being the least predictive among the group of four RF-based SFs, RF::Cyscore still preserved the inherent learning capability and kept lifting performance persistently with more training data (Figures 9, 11 and 13, top row), which was not seen in classical SFs. Although RF::Cyscore performed far worse than MLR::Cyscore initially (e.g. Rp = 0.402 versus 0.444 and RMSE = 1.61 versus 1.57 at a cutoff of 0.4 under protein structure similarity), through learning it kept improving and surpassed MLR::Cyscore in Rp at a cutoff of 0.79 and in RMSE at a cutoff of 0.88. Their performance gap was widened when the full training set was exploited, on which RF::Cyscore managed to yield a sharp leap, leading to much better performance (Rp = 0.513 versus 0.448, Rs = 0.515 versus 0.479, RMSE = 1.53 versus 1.59). Thanks to the learning capability, even this least predictive ML-based SF could improve Rp by 0.111 and reduce RMSE by 0.08. Moreover, low-quality structural and interaction data, referring to those samples in the PDBbind general set but not in the refined set, were previously found to improve the scoring power of RF-based SFs [20]. Compounded with this beneficial effect contributed by low-quality samples, the performance gap between RF-based and classical SFs is likely to be amplified.

In contrast, classical SFs lack such learning capability and thus their performance curves nearly stay flat (Figure 9, top row), as also seen recently [3–8]. For example, at the two ends of protein structure similarity cutoff (i.e. 0.4 and 1.0), the Rp varied slightly from 0.486 to 0.492 for MLR::Xscore, from 0.513 to 0.519 for MLR::Vina, and from 0.444 to 0.448 for MLR::Cyscore. Surprisingly, unlike minor improvements were observed in Rp, their RMSE even worsened a little bit, from 1.53 to 1.56 for MLR::Xscore and from 1.57 to 1.59 for MLR::Cyscore (Figure 13, top row). In Shen *et al.*’s study, the performance of Cyscore, ASP@GOLD, Alpha-HB@MOE and GBVIWSA-dG@MOE did not change much with increasing similarity either [6]. In Su *et al.*’s study, ChemScore, ASP and X-Score were found to be basically insensitive to the similarity between the training set and the test set or the sample size of the training set [7]. This category of SFs, owing to insufficient model complexity with few parameters and imposition of a fixed functional form, could not benefit from more training data, even those that are most relevant to the test set, confirming again that classical SFs are unable to exploit large volumes of structural and interaction data [5, 7]. This is, in our opinion, a critical disadvantage given the continuous growth of structural and interaction data in the future, which will further magnify the performance gap between ML-based and classical SFs (Figure 1). To our surprise, such a disadvantage was mistakenly regarded as an advantage by others who claimed that the performance of X-Score is relatively stable no matter what training data are used to fit the weights of its energy terms [3], and a classical SF may be more suitable when the target is completely novel [6]. Indeed, the performance of classical SFs is insensitive to training set composition (or in other terms, stable), but it does not imply a better performance than ML-based SFs (we will soon see that the opposite is true in most cases in the next subsection), and it is arduous to define ‘completely novel’ given that a large training set may inevitably contain any degree of similarity to the test set [4]. We will revisit this argument after we discuss the limitations of the CASF benchmarks later.

### Low proportion of dissimilar training complexes required by ML-based SFs to outperform classical SFs

Evaluated in Rp on CASF-2016 (Figure 8, top left subfigure), RF::Xscore was not able to surpass MLR::Xscore, the best performing classical SF among the three, until the protein structure similarity cutoff reached 0.99. The same is true on CASF-2013 [8] as well as on CASF-2007 where RF-Score was unable to outperform X-Score until the cutoff reached 0.98 [3]. Hence it is not surprising for Li and Yang to assert that ML-based SFs did not outperform classical SFs after removal of training complexes highly similar to the test set. However, this assertion does not hold when considering the RMSE metric where RF::Xscore produced lower values than MLR::Xscore from a cutoff of 0.89 onwards (Figure 14, top row). Nor does it hold upon substituting ligand fingerprint similarity (or pocket topology dissimilarity) where RF::Xscore started to overtake MLR::Xscore when the cutoff reached just 0.80 on CASF-2016 (Figure 8, top center subfigure) and 0.86 on CASF-2013 [8]. Nor does this assertion hold on Blind-2018 (Figure 9, top left subfigure) either where the best performing classical SF turned out to be MLR::Vina instead, which was surpassed by its RF variant RF::Vina at a cutoff of just 0.48. Now it is clear that this assertion is restrictive on three conditions: the Rp or Rs metric, protein similarity and CASF benchmarks have to be employed. Violating any condition voids the assertation.

**Figure 15:**
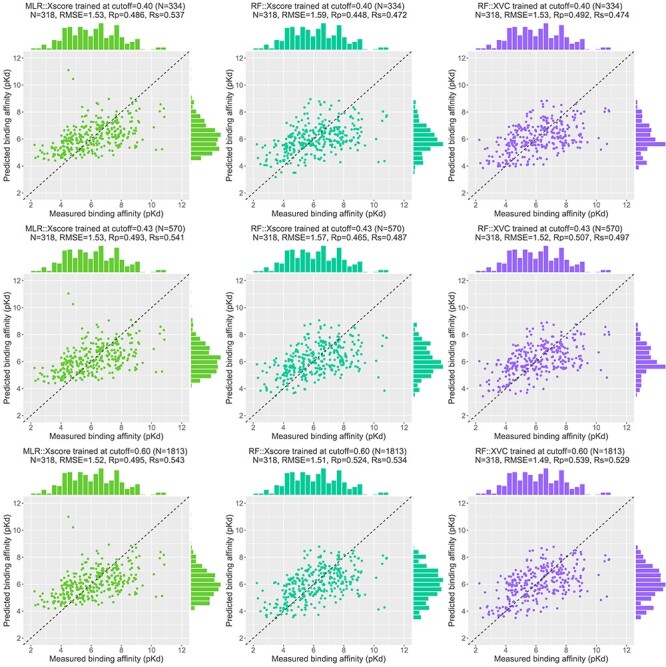
Scatter plots of predicted and measured binding affinities on Blind-2018 (*N* = 318 test complexes). Three SFs are compared: MLR::Xscore (left column), RF::Xscore (center column) and RF::XVC (right column), trained at three different cutoffs: 0.40 (1st row), 0.43 (2nd row) and 0.60 (3rd row), corresponding to 334, 570 and 1813 training complexes, respectively. The cutoff 0.40 represents the initial low end where MLR::Xscore resulted in a lower RMSE than RF::Xscore and RF::XVC. The cutoff 0.43 is the crossing point where RF::XVC started to generate lower RMSE values than MLR::Xscore. The cutoff 0.60 is another crossing point where RF::Xscore started to outperform MLR::Xscore in terms of RMSE.

As proposed previously [4], an alternative approach is to investigate the performance of SFs against the corresponding number of training complexes rather than the underlying cut-off value. This approach had been adopted in subsequent studies [5, 6, 8]. We therefore plotted the second row of Figure 9, making explicit the number of complexes in each training set, to evidence that ML-based SFs only required a small part of the full training set to outperform the classical SFs. MLR::Vina obtained the highest Rp among the three classical SFs considered, yet it was outperformed by RF::Vina trained on 1159 (28% of the full training set), 271 (7%) and 617 (15%) complexes dissimilar to the test set under the protein structure similarity, ligand fingerprint similarity and pocket topology dissimilarity metrics, respectively. Likewise, the second best performing classical SF, MLR::Xscore, was outperformed by RF::Xscore trained on 976 (23%), 271 (7%) and 504 (12%) complexes. These results are remarkable in the sense that the two comparing SFs utilize the same set of features and differentiate each other by their employed regression algorithm only. This trend is more obvious for RF::XVC. By integrating the features from X-Score, Vina and Cyscore, it required just 703 (17%), 271 (7%) and 558 (13%) dissimilar training complexes to surpass MLR::Vina, and 334 (8%), 271 (7%) and 529 (13%) to surpass MLR::Xscore. In terms of RMSE (Figure 13), MLR::Xscore obtained the lowest RMSE among the three classical SFs, but it was overtaken by RF::Xscore trained on 1813 (44%), 292 (7%), 656 (16%) dissimilar complexes, and by RF::XVC trained on 570 (14%), 271 (7%), 529 (13%) dissimilar complexes (Figure 15). Taken together, these results reveal for the first time that the proportion (i.e. 8%) of training complexes dissimilar to the test set required by ML-based SFs to outperform X-Score in Rp turns out to be far lower than what have been reported recently, i.e. 32% on CASF-2007 [5], 45% on CASF-2013 [8], and 63% on CASF-2016 (Figure 8).

Recall that the TM-score magnitude relative to random structures is not dependent on the protein’s size. Structures with a score higher than 0.5 assume generally the same fold [10]. Coincidently, RF::Vina obtained higher Rp values than MLR::Vina starting at a cutoff of 0.48. All the three classical SFs were overtaken by RF::XVC starting at a cutoff of 0.44 in terms of Rp and 0.43 in terms of RMSE. Thus, the proteins of these training samples do not assume the same fold, yet they contributed to the superior performance of ML-based SFs.

### Greater contributions of similar training complexes than dissimilar ones

We now explore a different scenario, represented by the bottom two rows of Figure 9, where the training set was originally composed of complexes highly similar to those in the test set only and then regularly enlarged to include dissimilar complexes as well (i.e. the sd direction). The Rp curves of RF::Xscore, RF::Vina and RF::Cyscore are always above that of their respective classical SF, not even to mention their hybrid variants RF::XVC and XGB::XVC. Likewise, these ML-based SFs always generated lower RMSE values than their corresponding classical counterpart (Figure 13) regardless of either the cutoff or the similarity metric. This is also true on CASF-2016 (Figure 12). These findings constitute a strong conclusion that under no circumstances did any of the classical SFs outperform their ML variant. This was one of the major conclusions by Li *et al.* on CASF-2007 [5] and by Sze *et al.* on CASF-2013 [8], and now it is deemed generalizable to the larger CASF-2016 and Blind-2018 benchmarks being investigated here. Consistently, most of the 28 ML-based SFs benchmarked on CASF-2007 also showed a remarkably better performance than their corresponding classical ones [6].

Consistent with common belief and hereby validated again, training complexes similar to those in the test set contribute appreciably more to the scoring power of ML-based SFs than dissimilar complexes. For example, RF::XVC yielded Rp = 0.654, Rs = 0.623, RMSE = 1.40 when trained on 628 complexes (cutoff = 0.99 in the sd direction) comprising proteins similar to the test set (Figure 9, bottom left subfigure), versus Rp = 0.515, Rs = 0.511, RMSE = 1.50 when the same SF was trained on 703 complexes (cutoff = 0.84 in the ds direction) comprising dissimilar proteins (Figure 9, second row left subfigure). This result also rationalizes the sharp leap phenomenon observed in ML-based SFs only.

Unlike in the ds direction where the peak performance for ML-based SFs was reached by exploiting the full training set of 4154 complexes, here in the sd direction the peak Rp performance was achieved at a cutoff of 0.43 (corresponding to 3584 complexes) for RF::XVC, 0.53 (2629 complexes) for RF::Vina, and 0.88 (1651 complexes) for RF::Xscore. Such peaks were observed under all the three similarity metrics as well as on all the three CASF benchmarks [5, 8] (Figures 8, 10 and 12) and Blind-2018 (Figures 9, 11 and 13). Their occurrence is likely owing to a compromise between the size of the training set and its relevance to the test data: encompassing additional complexes dissimilar to the test set beyond a certain threshold of similarity cutoff would probably introduce noise. That said, the performance variation between ML-based SFs trained on a subset spawn from an optimal cutoff and those trained on the full set is marginal. For instance, the Rp obtained by RF::XVC trained on the full set was 0.668, just marginally lower than its peak performance of 0.675 (for RMSE it was 1.32 with the full set of 4154 complexes, also only marginally worse than the best performance of 1.30 obtained at a cutoff of 0.43 with 3584 complexes), consistent with the recent conclusion that the addition of more dissimilar proteins into the training set does not clearly influence the final performance too much when there are enough training samples [6]. On the other hand, training ML-based SFs on the full set of complexes, despite being slightly less predictive than training on a prudently selected subset, has the hidden advantage of a broader applicability domain, hinting that such models should predict better on more diverse test sets containing protein families absent in the Blind-2018 benchmark. Besides, this simple approach of employing the full set for training does not bother to search for the optimal cut-off value, which does not seem an easy task. Failing that would probably incur a suboptimal performance than simply utilizing the full set.

### Feature hybridizing capability of ML-based SFs as another advantage over classical SFs

Thanks to the nonlinear nature of ML models, it is not difficult to hybridize features from multiple existing SFs, even those having distinct physiochemical semantics (such as geometric features, physical force field energy terms and pharmacophore features [21]), thus offering an opportunity to explore various combinations and construct an optimal SF. RF::XVC was built by combining features of X-Score, Vina and Cyscore, exhibiting better performance than their individual RF-based SF. It obtained an Rp of 0.668 on Blind-2018 (Figure 9), higher than that of RF::Xscore (0.646), RF::Vina (0.649), and RF::Cyscore (0.513). Likewise, RF::XVC achieved an RMSE of 1.32 (Figure 13), lower than that of RF::Xscore (1.34), RF::Vina (1.35) and RF::Cyscore (1.53). It also performed the best on CASF-2016 (Figures 8, 10 and 12) and CASF-2013 [8]. Likewise, RF-Score-v3 [18] was built by hybridizing the original pairwise descriptors of RF-Score (i.e. occurrence count of intermolecular contacts between elemental atom type pairs) with Vina empirical energy terms, which are two very different types of features. It was shown to perform better than RF-Score and RF::Vina on CASF-2007 [5]. RF-Score-v2 needed approximately 800 training samples to outperform classical SFs in the ds direction, but after hybridizing with GalaxyDock-BP2-Score, Smina or X-Score, only around 550, 600 or 700 training samples were needed [6]. More examples include NNscore 1.0 and NNscore 2.0, which required 40% fewer training samples to outperform classical SFs after hybridization. In the sd direction, in order to reach Rp ≥ 0.65, around 250, 300 and 250 training samples were compulsory for NNscore1.0, NNscore2.0 and RF-Score-v2, respectively, whereas only 200 training samples were mandatory for hybrid SFs [6]. In a comparative assessment of 12 ML-based SFs on CASF-2013, 108 terms from RF-Score, BALL, X-Score and SLIDE were used initially, which were subsequently reduced to just 17 terms with principal component analysis. Three resultant SFs, RF@ML, BRT@ML and kNN@ML, obtained a Rp of 0.704, 0.694 and 0.672, respectively, higher than 0.614 achieved by the best of 20 classical SFs [22]. Although hybrid SFs do not necessarily outperform single ones, this available capability provides a chance to search for the best mixture of descriptors that would lead to peak performance. For instance, hybridizing the pairwise descriptors of RF-Score into RF::XVC could further reduce the required percentage (currently 8%) of dissimilar training complexes for ML-based SFs to surpass classical SFs.

In contrast, feature hybridization is tough for classical SFs which rely on linear regression. The energy term accounting for hydrogen bonding is present in X-Score, Vina and Cyscore, but is implemented differently. How would one devise MLR::XVC remains to be explored. Even more challenging is to merge two streams of features in distinct scales or units, such as merging the atom type pair occurrence count (without unit) used by RF-Score and the energy terms (in kcal/mol units) used by Vina.

### Comparison between different ML regression methods

**Figure 16:**
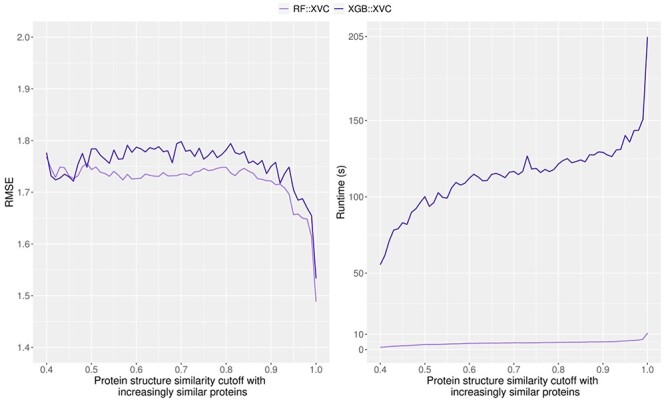
RMSE performance (left) and training time (right) of RF::XVC and XGB::XVC on the CASF-2016 benchmark when they were calibrated on different protein structure similarity cutoffs.

Both RF and XGBoost are tree-based ML algorithms. Both RF::XVC and XGB::XVC were demonstrated to possess learning capability and keep improving performance with training set size on Blind-2018 (Figure 9) and CASF-2016 (Figure 8) in the ds direction, finally yielding a sharp leap. Here RF::XVC outperformed XGB::XVC under most settings, probably due to suboptimal tuning of the hyperparameters of XGB. As a reference, an XGB-based SF utilizing the same set of descriptors as by RF-Score-v3 was found to marginally outperform the latter on CASF-2007 [5]. Regarding algorithmic efficiency, the training time required by XGB::XVC was 19–50 times as much as that required by RF::XVC (Figure 16).

The excellent performance of RF over other ML algorithms was also observed in two recent studies investigating the influence of data similarity. A comparative assessment of 25 commonly used SFs showed that GBDT and RF produced the best Rp values for most of the SFs on CASF-2007, performing better than ET, XGBoost, SVR and kNN [6]. Removing structurally redundant samples from the training set at a threshold of 95% on CASF-2016 effectively inhibited the learning capability of BRR, DT, kNN, MLP and SVR except RF [7]. More examples include RF@ML, which outperformed all other 11 ML algorithms on CASF-2013 [22].

Given that extensive efforts are usually compulsory for fine-tuning the hyperparameters of deep learning (DL) algorithms, it is computationally prohibitive for us to conduct the systematic trials presented here. There are evidences showing that DL-based SFs were not always more predictive than those based on more established ML methods [1]. That said, it is worthwhile to inspect deeper into this aspect in the future, since there are also evidences showing that DL is competing RF in computational docking [23].

### Limitations of the CASF benchmarks

Six related studies [3–8] all employed the CASF benchmarks to investigate the impact of data similarity. These benchmarks were formed by clustering a selected subset of complexes in the PDBbind refined set according to 90% similarity in protein sequences, and only the clusters containing more than five members (for CASF-2016) or three members (for CASF-2013 and CASF-2007) were considered. By construction, the CASF benchmarks are affected by protein homology bias in that only popular proteins having sufficient number of high-quality crystal structures are included for benchmarking purpose, leaving out a large amount of unpopular proteins (e.g. only 285 test set complexes picked from 57 clusters of CASF-2016 versus 3772 training set complexes left in the refined set). By comparing the results on CASF-2013, CASF-2016 and Blind-2018, it is clear that the CASF benchmarks overestimate the performance of SFs, whether these are ML-based or not. For example, RF::XVC achieved Rp = 0.714, 0.754 and 0.668 on the three benchmarks, respectively. A decline of 0.086 in Rp ensued when transiting the benchmark from CASF-2016 to Blind-2018. Likewise, MLR::Xscore obtained Rp = 0.622, 0.647 and 0.492, respectively, equivalent to a decline of 0.155, almost twice as much. Albeit the Rp performance of both categories of SFs being overestimated by CASF, some authors only saw the need of employing new benchmarks for ML-based SFs [7].

Although both categories of SFs suffered from performance degradation on the more realistic Blind-2018 benchmark, the degree is different (Rp dropped by 0.155 for MLR::Xscore versus just 0.086 for RF::XVC). This point can be revalidated from another perspective. The percentage of dissimilar training complexes required by RF::XVC to outperform X-Score was 45% on CASF-2013 and 63% on CASF-2016, far higher than just 8% on Blind-2018. This means X-Score performed unusually well on CASF, making it tough for ML-based SFs to excel. In fact, X-Score was the best performing classical SF on CASF-2016 and CASF-2013 and the second best on CASF-2007 (Figure 1). Hence the CASF benchmarks tend to overestimate the performance of classical SFs much more than that of ML-based SFs.

We think of two ways to circumvent the effect of overestimation brought by CASF. The first is to employ the Blind-2018 benchmark [1], which is by construction a blind test in that only structural and interaction data available until 2017 are used to build the SF that predicts the binding affinities of complexes released in 2018 as if these had not been measured yet. Thus this benchmark realistically imitates a prospective scenario without introducing artificial factors that specifically harden the challenge for either category of SFs. Another way is to embrace the concept of soft overlap [7], with which training sets were compiled from the PDBbind refined set by removing redundant samples under four different similarity thresholds of 80%, 85%, 90% and 95%. However, we think that these nonredundant training sets are of little use because five of the six tested ML-based SFs all outperformed the three comparing classical SFs. The authors should instead provide a training set where ML-based SFs fail to outperform classical ones, but such a training set is understandably difficult to provide because when the similarity threshold decreases, the sample size decreases too, down below a so-called ‘healthy’ size of around 2100 complexes, which corresponds to the number of complexes of the nonredundant set compiled from the smallest refined set (i.e. v2016) under the lowest threshold (i.e. 80%). This issue can be addressed either by discarding CASF-2016 as the test set, which these authors had not attempted, or by shrinking the sample size (or the similarity threshold) to a point where the niche of classical SFs could be appreciated [4]. Regarding the latter, remind that RF::XVC required merely 334, 271 and 529 dissimilar complexes to surpass MLR::Xscore under the three similarity metrics. Should these nonredundant sets be useful for comparing ML-based and classical SFs, their sample size probably has to be reduced to below 271, which is about a half quarter of the healthy sample size they wanted to preserve. These sets, nevertheless, can be useful for examining the learning capability of ML-based SFs with increasingly similar samples.

### Inevitability of similarity contained in a large dataset

It was claimed that ML-based SFs are unlikely to give reliable predictions for completely novel targets [6], or may be less effective when dealing with structurally novel targets or ligand molecules [7], but no formal definition for ‘novel’ was given. Here we come up with two possible definitions. The first is to set up a fixed similarity threshold, below which complexes are considered to be novel. Recall that at a protein structure cutoff of 0.40 (a TM-score below 0.5 assumes generally distinct folds), which corresponds to 334 (8%) training complexes, RF::XVC already generated a higher Rp than MLR::Xscore (Rp = 0.492 versus 0.486 on Blind-2018). Hence the similarity threshold used to define novel has to be set to a rather low value in order for the two claims to hold true. The second is to follow the old-fashioned approach of leave-cluster-out cross-validation, where the whole dataset is first clustered by 90% sequence identity and then the clusters are split to form nonoverlapping training and test sets [24]. The test complexes whose proteins do not fall in any of the training clusters are considered as novel. By leaving one cluster out, an early study showed that the Rp averaged across 26 clusters was 0.493 for MLR::Cyscore, 0.515 for RF::CyscoreVina and 0.545 for RF::CyscoreVinaElem [19], indicating that ML-based SFs hybridizing Cyscore features with those of Vina and RF-Score still outperformed Cyscore itself, rendering the two claims wrong. To make them right, one may further harden the challenge by leaving two or three clusters out. No matter which definition, overoptimizing the training set composition to artificially toughen the benchmark without considering the full spectrum of complexes to be found in a prospective environment can lead to false confidence. Adding training complexes that are similar to the test set to some extent actually helps mimic a real scenario, as they represent the overall characteristics of the existing available samples.

Recently we asserted that a large dataset may inevitably contain proteins with any degree of similarity to those in the test set [4]. Here we provide concrete statistics to support this assertion. A TM-score below 0.17 corresponds to randomly chosen unrelated proteins [9], but neither any of the 3772 training complexes nor any of the 4154 training complexes has a test set protein structure similarity below 0.17 on CASF-2016 and Blind-2018, respectively, suggesting that the training and test proteins are unavoidably related. When relaxing the TM-score filter to no higher than 0.5, only 1562 (out of 3772) and 1343 (out of 4154) training complexes can be assumed having different folds [10], still less than half of the full set. Even though one can find a training complex that is sufficiently dissimilar to the test set under all the three similarity metrics investigated in this study (or the 3-in-1 combined metric [7]), there may exist an extra metric under which this particular training sample shows a high similarity. Therefore, it is inevitable for a large dataset to exhibit a certain degree of similarity to the test set. In this context, it is meaningless to argue whether or not the training set contains samples similar to the test set, or whether overestimation occurs. What is really meaningful is whether the SFs can make the best use of such similarity inherently contained in the training set. Nothing is wrong if one can make a reasonable prediction based on the available information on similar samples [7]. The statement that the performance of ML-based SFs is overestimated due to the presence of training complexes similar to the test set [6] can be alternatively interpreted as that the performance of ML-based SFs is underestimated due to the absence of similar samples in those artificially created training sets that specifically harden the challenge for ML-based SFs.

So now the question becomes, which category of SFs can make the best use of the similarity or the dissimilarity contained in the dataset and thus should be preferred? The answer is obvious. Although ML-based SFs did not stand out significantly when calibrated on a training set with 80% similarity to the test set (i.e. the low end) [7], they still performed better than classical counterparts. The RF, kNN, MLP, BRR and L-SVR models obtained an Rp of about 0.703, 0.683, 0.678, 0.665 and 0.645, respectively, consistently (though not significantly) higher than 0.625, 0.620 and 0.480 obtained by X-Score^HM^, ChemScore and ASP. Should this represent a prospective scenario where only a training set with 80% similarity to the target set of interest is available, ML-based SFs would still be preferred. On the other hand, classical SFs have been consistently found to suffer from early stagnation of performance when given similar complexes for regression [3–8]. Again, being stable is hardly equivalent to being superior. Performance stability does not guarantee performance superiority.

## Conclusions

For the first time we have revealed that ML-based SFs trained on just 8% training complexes dissimilar to the test set already outperformed the representative classical SF X-Score on a blind benchmark that emulates the real process of prospective prediction of binding affinity. This proportion is substantially lower than what was previously reported on the CASF benchmarks [5, 8], suggesting that the scoring power of classical SFs is overestimated on CASF. When the benchmark was transited to Blind-2018, the Rp performances of both categories of SFs were downgraded, but more severely for classical SFs. The performance of ML-based SFs was claimed to be overestimated in the existence of similarity, but the same statement should be reinterpreted as that these are underestimated in the absence of similar samples on those artificially created training sets that abandon the full spectrum of complexes to be found in a prospective campaign. Adding training complexes that are similar to the test set to some extent effectively facilitates the imitation of a real scenario, as they represent the general characteristics of the existing materials accessible at hand. Given the unavoidability of any degree of similarity incorporated in a large dataset, the criteria for SF selection are not performance stability but superiority, i.e. whether the SF can make the best use of all available information in order to make a reasonable prediction of the binding affinity of the target complexes of interest in a prospective situation. Considering the continuous growth of structural and interaction data in the future, the development of ML-based SFs is becoming appealing, as the performance gap to classical SFs will be further broadened.

The contributions of this study are manifold. We compiled the Rp performances of 70 unique SFs benchmarked on three versions of CASF (Supplementary Tables S1–S3) and plotted Figure 1 to vividly show that ML-based SFs surpass classical counterparts by a large margin. We reviewed a list of six related studies (Supplementary Table S4), drew a workflow chart for investigating the impact of training-test set similarity (Figure 2), and showcased three examples of misalignment caused by the all-chains-against-all-chains approach (Figures 4 and 5, Supplementary Materials S1 and S2) employed in four of the six studies. We illustrated the distribution of training complexes over the protein structure similarity cutoff is skewed toward the range of (0.99, 1] (Figures 6 and 7), where sharp leaps in scoring power of ML-based SFs are reasonably anticipated (Figures 8 and 9). The unique capabilities of learning and feature hybridization were demonstrated to be advantages of ML-based SFs, which classical SFs lack, ending up with early stagnation of performance. Lastly, we have released free software code and necessary data at https://github.com/cusdulab/MLSF for interested readers to rapidly reproduce the results presented in this study and to make an extended analysis on their own benchmark, e.g. Blind-2020, to be constructed in a similar way as Blind-2018.

**Key Points**

- The influence of data similarity on the scoring power of three widely used classical SFs and five ML-based variants was assessed on two benchmarks, including a blind test mimicking a real scenario of prospective prediction of binding affinities.
- ML-based SFs trained on just 8% complexes dissimilar to the test set already outperform classical SFs on a blind benchmark. This percentage is far lower than what was required on any of the three CASF benchmarks.
- The capabilities of learning and feature hybridization are two advantages of ML-based SFs over classical ones. Their performance gap will be amplified given the continuous growth of structural and interaction data.
- The CASF benchmarks tend to overestimate the performance of SFs, whether these are ML-based or not. But the degree of overestimation is higher for classical SFs because a more significant performance degradation was observed when transiting to a blind benchmark.
- A large dataset may inevitably contain proteins with any degree of similarity to those in the test set. The criteria for SF selection are not performance stability but superiority. Nothing is wrong if one can make a reasonable prediction by exploiting data similarity.

## Supplementary Material

MLSFTOCDTTTSAOCCOABB-SI20210423_bbab225

## Notes

**Hongjian Li** received his PhD degree from Chinese University of Hong Kong in 2015. His research focuses on computer-aided drug design, scoring function development, virtual screening and machine learning. He is the lead developer of the molecular docking software idock, the web server platform istar, the WebGL visualizer iview and the scoring function RF-Score-v3. More information can be found at his website at https://github.com/HongjianLi.

**Gang Lu** is a senior research fellow in the School of Biomedical Sciences, Chinese University of Hong Kong. He established the bioinformatics unit and currently engages in drug screening, drug repositioning, neural regenerative medicine, cell therapeutic strategies and disease diagnosis.

**Kam-Heung Sze** is an engineer in the Bioinformatics Unit, Hong Kong Medical Technology Institute. She works on computer-aided drug design, especially data collection, software evaluation, script writing and computational pipeline automation.

**Xianwei Su** was an honorary research associate of the Chinese University of Hong Kong. His research interests lie in liquid biopsy, biomarker identification, circulating miRNA, dendritic dell maturation and aneurysmal subarachnoid hemorrhage.

**Wai-Yee Chan** is the Director of the CUHK-SDU Joint Laboratory on Reproductive Genetics. He was the Director of the School of Biomedical Sciences and the Pro Vice-Chancellor/Vice President of the Chinese University of Hong Kong. His expertise is in the molecular genetics of human reproductive and endocrine disorders, biochemical genetics of inborn errors of copper and zinc metabolism, functional genomics and epigenomics.

**Kwong-Sak Leung** was a Chair Professor of Computer Science and Engineering in the Chinese University of Hong Kong. He is also a fellow of HKIE, a member of IET and ACM, a senior member of IEEE and a chartered engineer. His research interests are in the areas of knowledge engineering, bioinformatics, soft computing, genetic algorithms and programming, automatic knowledge acquisition, fuzzy logic applications and AI architecture.
