# Supplementary material for: Machine-learning scoring functions trained on complexes dissimilar to the test set already outperform classical counterparts on a blind benchmark
Source: Brief Bioinform. 2021 Jun 24;22(6):bbab225. doi: 10.1093/bib/bbab225 (PMC8575004; doi:10.1093/bib/bbab225)
Supplement: MLSFTOCDTTTSAOCCOABB-SI20210423_bbab225 [file mlsftocdtttsaoccoabb-si20210423_bbab225.docx]

| Machine-learning scoring functions trained on complexes dissimilar to the test set already outperform classical counterparts on a blind benchmark – Supplementary information  Hongjian Li^1,2^, Gang Lu^1,*^, Kam-Heung Sze^2^, Xianwei Su^2,1^, Wai-Yee Chan^1^, Kwong-Sak Leung^3^  ^1^CUHK-SDU Joint Laboratory on Reproductive Genetics, School of Biomedical Sciences, Chinese University of Hong Kong, Hong Kong.  ^2^Bioinformatics Unit, SDIVF R&D Centre, Hong Kong.  ^3^Department of Computer Science and Engineering, Chinese University of Hong Kong, Hong Kong.  *To whom correspondence should be addressed. |
| --- |

# Table S1. Rp performance of 35 SFs on CASF-2007.

# Table S2. Rp performance of 29 SFs on CASF-2013.

# Table S3. Rp performance of 43 SFs on CASF-2016.

# Table S4. Recent studies investigating the impact of data similarity on the scoring performance of SFs.

# Table S5. Rp performance of 11 SFs on Blind-2018.

# Table S6. Molecular features from X-Score, Vina and Cyscore.

# Text S1. Output of TM-align for aligning 3UOD chain A to 3E5A chain A.

# Text S2. Output of TM-align for aligning 3UOD chain A to 3E5A chain B.

# Text S3. Output of MM-align for aligning 3UOD to 3E5A.

**Table S1.** Rp performance of 35 SFs on CASF-2007. The Excel version of this table is available at <https://github.com/cusdulab/MLSF>.

| Publication date | Scoring function | Regression model | Rp |
| --- | --- | --- | --- |
| 2019-08-26 | Boyles et al. | RF | 0.836 |
| 2019-06-18 | AGL-Score | GBDT | 0.830 |
| 2019-03-14 | XGB-Score | XGBoost | 0.806 |
| 2019-01-28 | EIC-Score | GBDT | 0.817 |
| 2018-11-02 | PotentialNet | GCN | 0.822 |
| 2018-01-08 | TopBP | GBDT & CNN | 0.827 |
| 2017-07-27 | TNet-BP | CNN | 0.826 |
| 2017-06-30 | RI-Score | RF | 0.803 |
| 2017-06-29 | T-Bind | GBDT | 0.818 |
| 2017-04-03 | FFT-BP | GBDT | 0.800 |
| 2016-05-05 | CSM-Lig | GP | 0.751 |
| 2015-02-23 | BsN-Score | NN | 0.816 |
| 2014-08-25 | RF::XR | RF | 0.806 |
| 2014-02-21 | Cyscore | Linear | 0.657 |
| 2014-02-16 | RF-Score v2 | RF | 0.803 |
| 2011-12-01 | CScore | NN | 0.801 |
| 2010-03-17 | RF-Score v1 | RF | 0.776 |
| 2009-06-04 | AutoDock Vina | Linear | 0.596 |
| 2009-04-09 | X-Score | Linear | 0.644 |
| 2009-04-09 | DrugScoreCSD | Linear | 0.569 |
| 2009-04-09 | SYBYL::ChemScore | Linear | 0.555 |
| 2009-04-09 | DS::PLP1 | Linear | 0.545 |
| 2009-04-09 | GOLD::ASP | Linear | 0.534 |
| 2009-04-09 | SYBYL::G-Score | Linear | 0.492 |
| 2009-04-09 | DS::LUDI3 | Linear | 0.487 |
| 2009-04-09 | DS::LigScore2 | Linear | 0.464 |
| 2009-04-09 | GlideScore-XP | Linear | 0.457 |
| 2009-04-09 | DS::PMF | Linear | 0.445 |
| 2009-04-09 | GOLD::ChemScore | Linear | 0.441 |
| 2009-04-09 | by NHA | Linear | 0.431 |
| 2009-04-09 | SYBYL::D-Score | Linear | 0.392 |
| 2009-04-09 | DS::Jain | Linear | 0.316 |
| 2009-04-09 | GOLD::GoldScore | Linear | 0.295 |
| 2009-04-09 | SYBYL::PMF-Score | Linear | 0.268 |
| 2009-04-09 | SYBYL::F-Score | Linear | 0.216 |

**Table S2.** Rp performance of 29 SFs on CASF-2013. The Excel version of this table is available at <https://github.com/cusdulab/MLSF>.

| Publication date | Scoring function | Regression model | Rp |
| --- | --- | --- | --- |
| 2019-08-26 | Boyles et al. | RF | 0.780 |
| 2019-06-18 | AGL-Score | GBDT | 0.792 |
| 2019-01-28 | EIC-Score | GBDT | 0.774 |
| 2018-01-08 | TopBP | GBDT & CNN | 0.808 |
| 2017-06-30 | RI-Score | RF | 0.782 |
| 2017-06-29 | T-Bind | GBDT | 0.767 |
| 2016-05-05 | CSM-lig | GP | 0.800 |
| 2015-07-16 | RF@ML | RF | 0.704 |
| 2014-04-07 | X-ScoreHM | Linear | 0.614 |
| 2014-04-07 | ΔSAS | Linear | 0.606 |
| 2014-04-07 | ChemScore@SYBYL | Linear | 0.592 |
| 2014-04-07 | ChemPLP@GOLD | Linear | 0.579 |
| 2014-04-07 | PLP1@DS | Linear | 0.568 |
| 2014-04-07 | G-Score@SYBYL | Linear | 0.558 |
| 2014-04-07 | ASP@GOLD | Linear | 0.556 |
| 2014-04-07 | ASE@MOE | Linear | 0.544 |
| 2014-04-07 | ChemScore@GOLD | Linear | 0.536 |
| 2014-04-07 | D-Score@SYBYL | Linear | 0.526 |
| 2014-04-07 | Alpha-HB@MOE | Linear | 0.511 |
| 2014-04-07 | LUDI3@DS | Linear | 0.487 |
| 2014-04-07 | GoldScore@GOLD | Linear | 0.483 |
| 2014-04-07 | Affinity-dG@MOE | Linear | 0.482 |
| 2014-04-07 | LigScore2@DS | Linear | 0.456 |
| 2014-04-07 | GlideScore-SP | Linear | 0.452 |
| 2014-04-07 | Jain@DS | Linear | 0.408 |
| 2014-04-07 | PMF@DS | Linear | 0.364 |
| 2014-04-07 | GlideScore-XP | Linear | 0.277 |
| 2014-04-07 | London-dG@MOE | Linear | 0.242 |
| 2014-04-07 | PMF@SYBYL | Linear | 0.221 |

**Table S3.** Rp performance of 43 SFs on CASF-2016. The Excel version of this table is available at <https://github.com/cusdulab/MLSF>.

| Publication date | Scoring function | Regression model | Rp |
| --- | --- | --- | --- |
| 2019-10-22 | ∆vinaXGB | XGB | 0.796 |
| 2019-08-26 | Boyles et al. | RF | 0.821 |
| 2019-06-18 | AGL-Score | GBDT | 0.833 |
| 2019-01-28 | EIC-Score | GBDT | 0.825 |
| 2018-04-14 | Affi & Al-Sadek | RF | 0.824 |
| 2018-01-08 | TopBP | GBDT & CNN | 0.861 |
| 2018-01-08 | K_DEEP_ | CNN | 0.820 |
| 2017-07-27 | TNet-BP | CNN | 0.810 |
| 2017-06-30 | RI-Score | RF | 0.815 |
| 2018-11-27 | ∆vinaRF20 | RF | 0.816 |
| 2018-11-27 | X-Score | Linear | 0.631 |
| 2018-11-27 | X-ScoreHS | Linear | 0.629 |
| 2018-11-27 | ∆SAS | Linear | 0.625 |
| 2018-11-27 | X-ScoreHP | Linear | 0.621 |
| 2018-11-27 | ASP@GOLD | Linear | 0.617 |
| 2018-11-27 | ChemPLP@GOLD | Linear | 0.614 |
| 2018-11-27 | X-ScoreHM | Linear | 0.609 |
| 2018-11-27 | AutoDock Vina | Linear | 0.604 |
| 2018-11-27 | DrugScore2018 | Linear | 0.602 |
| 2018-11-27 | DrugScoreCSD | Linear | 0.596 |
| 2018-11-27 | ASE@MOE | Linear | 0.591 |
| 2018-11-27 | ChemScore@SYBYL | Linear | 0.590 |
| 2018-11-27 | PLP1@DS | Linear | 0.581 |
| 2018-11-27 | ChemScore@GOLD | Linear | 0.574 |
| 2018-11-27 | G-Score@SYBYL | Linear | 0.572 |
| 2018-11-27 | Alpha-HB@MOE | Linear | 0.569 |
| 2018-11-27 | PLP2@DS | Linear | 0.563 |
| 2018-11-27 | Affinity-dG@MOE | Linear | 0.552 |
| 2018-11-27 | LigScore2@DS | Linear | 0.540 |
| 2018-11-27 | D-Score@SYBYL | Linear | 0.531 |
| 2018-11-27 | LUDI2@DS | Linear | 0.526 |
| 2018-11-27 | GlideScore-SP | Linear | 0.513 |
| 2018-11-27 | LUDI3@DS | Linear | 0.502 |
| 2018-11-27 | GBVI/WSA-dG@MOE | Linear | 0.496 |
| 2018-11-27 | LUDI1@DS | Linear | 0.494 |
| 2018-11-27 | GlideScore-XP | Linear | 0.467 |
| 2018-11-27 | Jain@DS | Linear | 0.457 |
| 2018-11-27 | LigScore1@DS | Linear | 0.425 |
| 2018-11-27 | PMF@DS | Linear | 0.422 |
| 2018-11-27 | GoldScore@GOLD | Linear | 0.416 |
| 2018-11-27 | London-dG@MOE | Linear | 0.405 |
| 2018-11-27 | PMF@SYBYL | Linear | 0.262 |
| 2018-11-27 | PMF04@DS | Linear | 0.212 |

**Table S4.** Recent studies investigating the impact of data similarity on the scoring performance of SFs, in ascending order of publication date.


| Publication date | Benchmarks | Scoring functions | Machine learning methods | Reference |
| --- | --- | --- | --- | --- |
| 2017-03-30 | CASF-2007 | X-Score, RF-Score | RF | (Li and Yang, 2017) |
| 2018-03-14 | CASF-2007 | X-Score, RF::X-Score, RF-Score, RF-Score-v3 | RF | (Li *et al.*, 2018) |
| 2019-10-15 | CASF-2007 | X-Score, Vina, Cyscore, RF::X-Score, RF::Vina, RF::Cyscore, RF-Score, RF-Score-v3, XGB-Score | RF, XGB | (Li *et al.*, 2019) |
| 2020-01-25 | CASF-2007 | 25 SFs, e.g. ASP@GOLD, Alpha-HB@MOE, GBVIWSA-dG@MOE, GalaxyDock-BP2-score, NNscore, RFScore-v2 | RF, ET, GBDT, XGB, SVR, kNN | (Shen *et al.*, 2020) |
| 2020-02-21 | CASF-2016 | ChemScore, ASP, X-Score | BRR, DT, kNN, MLP, L-SVR, RF | (Su *et al.*, 2020) |
| 2020-02-24 | CASF-2013 | X-Score, Vina, Cyscore, RF::Xscore, RF::Vina, RF::Cyscore, RF::XVC | RF | (Sze *et al.*, 2020) |
| Paper to be published | CASF-2016, Blind-2018 | X-Score, Vina, Cyscore, RF::Xscore, RF::Vina, RF::Cyscore, RF::XVC, XGB::XVC | RF, XGB | This paper |

References

Li,H. *et al.* (2019) Classical scoring functions for docking are unable to exploit large volumes of structural and interaction data. *Bioinformatics*, **35**, 3989–3995.

Li,H. *et al.* (2018) The impact of protein structure and sequence similarity on the accuracy of machine-learning scoring functions for binding affinity prediction. *Biomolecules*, **8**, 12.

Li,Y. and Yang,J. (2017) Structural and Sequence Similarity Makes a Significant Impact on Machine-Learning-Based Scoring Functions for Protein-Ligand Interactions. *J. Chem. Inf. Model.*, **57**, 1007–1012.

Shen,C. *et al.* (2020) Can machine learning consistently improve the scoring power of classical scoring functions? Insights into the role of machine learning in scoring functions. *Brief. Bioinform.*, **22**, 497–514.

Su,M. *et al.* (2020) Tapping on the Black Box: How Is the Scoring Power of a Machine-Learning Scoring Function Dependent on the Training Set? *J. Chem. Inf. Model.*, **60**, 1122–1136.

Sze,K.-H. *et al.* (2020) Influence of Data Similarity on the Scoring Power of Machine-learning Scoring Functions for Docking. In, *Proceedings of the 13th International Joint Conference on Biomedical Engineering Systems and Technologies - Volume 3: BIOINFORMATICS*. SciTePress, pp. 85–92.

**Table S5.** Rp performance of 11 SFs on Blind-2018. The Excel version of this table is available at <https://github.com/cusdulab/MLSF>.

| Publication date | Scoring function | Regression model | Rp |
| --- | --- | --- | --- |
| Paper to be published | XGB::XVC | XGB | 0.650 |
| Paper to be published | RF::XVC | RF | 0.668 |
| Paper to be published | RF::Cyscore | RF | 0.513 |
| Paper to be published | RF::Xscore | RF | 0.646 |
| Paper to be published | MLR::Cyscore | Linear | 0.448 |
| Paper to be published | MLR::Xscore | Linear | 0.492 |
| 2020-09-01 | RF::VinaElem | RF | 0.702 |
| 2020-09-01 | RF::Elem | RF | 0.669 |
| 2020-09-01 | RF::Vina | RF | 0.649 |
| 2020-09-01 | MLR::Vina | Linear | 0.519 |
| 2020-09-01 | AutoDock Vina | Linear | 0.500 |

**Table S6.** Molecular features from X-Score, Vina and Cyscore.

| Molecular feature | Explanation |
| --- | --- |
| Features from X-Score | |
| VDW | van der Waals interaction |
| HB | hydrogen bonding |
| HP | hydrophobic effect |
| HM | hydrophobic effect |
| HS | hydrophobic effect |
| RT | deformation penalty |
| Features from Vina | |
| Gauss1 | steric interaction |
| Gauss2 | steric interaction |
| Repulsion | steric interaction |
| Hydrophobic | hydrophobic interaction |
| Hydrogen bonding | hydrogen bonding |
| Nrot | conformational freedom of the ligand |
| Features from Cyscore | |
| Hydrophobic | hydrophobic free energy |
| Vdw | van der Waals interaction energy |
| HBond | hydrogen-bond energy |
| Ent | ligand's entropy |

**Text S1.** Output of TM-align for aligning 3UOD chain A to 3E5A chain A.

Name of Chain_1: 3UOD_protein_A.pdb

Name of Chain_2: 3E5A_protein_A.pdb

Length of Chain_1: 266 residues

Length of Chain_2: 264 residues

Aligned length= 262, RMSD= 1.42, Seq_ID=n_identical/n_aligned= 0.981

TM-score= 0.94695 (if normalized by length of Chain_1)

TM-score= 0.95392 (if normalized by length of Chain_2)

(You should use TM-score normalized by length of the reference protein)

(":" denotes aligned residue pairs of d < 5.0 A, "." denotes other aligned residues)

KKRQWALEDFEIGRPLGKGKFGNVYLAREKQSKFILALKVLFKAQLEKAGVEHQLRREVEIQSHLRHPNILRLYGYFHDATRVYLILEYAPLGTVYRELQKLSKFDEQRTATYITELANALSYCHSKRVIHRDIKPENLLLGSAGELKIADFGWSVHA--PSSRRDTLCGTLDYLPPEMIEGRMHDEKVDLWSLGVLCYEFLVGKPPFEANTYQETYKRISRVEFTFPDFVTEGARDLISRLLKHNPSQRPMLREVLEHPWITANSSK

.::::::::::::::::....:::::::::::::::::::::::::::::::::::::::::::::::::::::::::::::::::::::::::::::::::::::::::::::::::::::::::::::::::::::::::::::::::::::::: ::::: ::::::::::::::::::::::::::::::::::::::::::::::::::::::::::::::::::::::::::::::::::::::::::::::::::::

-KRQWALEDFEIGRPLGKGKFGNVYLAREKQSKFILALKVLFKAQLEKAGVEHQLRREVEIQSHLRHPNILRLYGYFHDATRVYLILEYAPLGTVYRELQKLSKFDEQRTATYITELANALSYCHSKRVIHRDIKPENLLLGSAGELKIADFGWSVHAPSSRRTL---CGTLDYLPPEMIEGRMHDEKVDLWSLGVLCYEFLVGKPPFEANTYQETYKRISRVEFTFPDFVTEGARDLISRLLKHNPSQRPMLREVLEHPWITANSSK

**Text S2.** Output of TM-align for aligning 3UOD chain A to 3E5A chain B.

Name of Chain_1: 3UOD_protein_A.pdb

Name of Chain_2: 3E5A_protein_B.pdb

Length of Chain_1: 266 residues

Length of Chain_2: 33 residues

Aligned length= 28, RMSD= 3.15, Seq_ID=n_identical/n_aligned= 0.036

TM-score= 0.08810 (if normalized by length of Chain_1)

TM-score= 0.36819 (if normalized by length of Chain_2)

(You should use TM-score normalized by length of the reference protein)

(":" denotes aligned residue pairs of d < 5.0 A, "." denotes other aligned residues)

KKRQWALEDFEIGRPLGKGKFGNVYLAREKQSKFILALKVLFKAQLEKAGVEHQLRREVEIQSHLRHPNILRLYGYFHDATRVYLILEYAPLGTVYRELQKLSKFDEQRTATYITELANALSYCHSKRVIHRDIKPENLLLGSAGELKIADFGWSVHAPSSRRDTLCG---TLDYLPPEMIEGRMH--DEKVDLWSLGVLCYEFLVGKPPFEANTYQETYKRISRVEFTFPDFVTEGARDLISRLLKHNPSQRPMLREVLEHPWITANSSK

.:::: :::::. .:: .:: :::::::::: .

------------------------------------------------------------------------------------------------------------------------------------------------------------------------SSYSYDAP----SDFINFSSGDT-QNI-DSWFEEKANL------------------------E----------------------------------------

**Text S3.** Output of MM-align for aligning 3UOD to 3E5A.

Protein 1:3UOD_prote Size= 266

Protein 2:3E5A_prote Size= 297 (TM-score is normalized by 297)

Aligned length= 262, RMSD= 1.48, TM-score=0.84974, ID=0.981

-------- rotation matrix to rotate Chain-1 to Chain-2 ------

i t(i) u(i,1) u(i,2) u(i,3)

1 56.3133376065 0.0594397133 -0.9952788890 0.0767258342

2 32.6125980339 -0.9915850712 -0.0500141828 0.1194053099

3 26.8871782297 -0.1150042042 -0.0831776092 -0.9898765167

(":" denotes the residue pairs of distance < 5.0 Angstrom)

kkrqwaledfeigrplgkgkfgnvylarekqskfilalkvlfkaqlekagvehqlrreveiqshlrhpnilrlygyfhdatrvylileyaplgtvyrelqklskfdeqrtatyitelanalsychskrvihrdikpenlllgsagelkiadfgwsvha--pssrrdtlcgtldylppemiegrmhdekvdlwslgvlcyeflvgkppfeantyqetykrisrveftfpdfvtegardlisrllkhnpsqrpmlrevlehpwitanssk---------------------------------

. ::::::::::::::::....:::::::::::::::::::::::::::::::::::::::::::::::::::::::::::::::::::::::::::::::::::::::::::::::::::::::::::::::::::::::::::::::::::::::: ::::: ::::::::::::::::::::::::::::::::::::::::::::::::::::::::::::::::::::::::::::::::::::::::::::::::::::

k-rqwaledfeigrplgkgkfgnvylarekqskfilalkvlfkaqlekagvehqlrreveiqshlrhpnilrlygyfhdatrvylileyaplgtvyrelqklskfdeqrtatyitelanalsychskrvihrdikpenlllgsagelkiadfgwsvhapssrrtl---cgtldylppemiegrmhdekvdlwslgvlcyeflvgkppfeantyqetykrisrveftfpdfvtegardlisrllkhnpsqrpmlrevlehpwitansskSSYSYDAPSDFINFSSGDTQNIDSWFEEKANLE

(Odd no chains of both proteins are in lower case and Even no chains of both proteins are in UPPER case)
